# Val-BoroPro Accelerates T Cell Priming via Modulation of Dendritic Cell Trafficking Resulting in Complete Regression of Established Murine Tumors

**DOI:** 10.1371/journal.pone.0058860

**Published:** 2013-03-12

**Authors:** Meghaan P. Walsh, Brynn Duncan, Shannon Larabee, Aviva Krauss, Jessica P. E. Davis, Yongzhi Cui, Su Young Kim, Martin Guimond, William Bachovchin, Terry J. Fry

**Affiliations:** 1 Pediatric Oncology Branch, Center for Cancer Research, National Cancer Institute, National Institutes of Health, Bethesda, Maryland, United States of America; 2 University of Montreal, Maisonneuve-Rosemont Hospital, Montreal, Quebec, Canada; 3 Tufts University Sackler School of Graduate Biomedical Sciences, Department of Biochemistry, Boston, Massachusetts, United States of America; University of Pécs Medical School, Hungary

## Abstract

Although tumors naturally prime adaptive immune responses, tolerance may limit the capacity to control progression and can compromise effectiveness of immune-based therapies for cancer. Post-proline cleaving enzymes (PPCE) modulate protein function through N-terminal dipeptide cleavage and inhibition of these enzymes has been shown to have anti-tumor activity. We investigated the mechanism by which Val-boroPro, a boronic dipeptide that inhibits post-proline cleaving enzymes, mediates tumor regression and tested whether this agent could serve as a novel immune adjuvant to dendritic cell vaccines in two different murine syngeneic murine tumors. In mice challenged with MB49, which expresses the HY antigen complex, T cell responses primed by the tumor with and without Val-boroPro were measured using interferon gamma ELISPOT. Antibody depletion and gene-deficient mice were used to establish the immune cell subsets required for tumor regression. We demonstrate that Val-boroPro mediates tumor eradication by accelerating the expansion of tumor-specific T cells. Interestingly, T cells primed by tumor during Val-boroPro treatment demonstrate increased capacity to reject tumors following adoptive transfer without further treatment of the recipient. Val-boroPro -mediated tumor regression requires dendritic cells and is associated with enhanced trafficking of dendritic cells to tumor draining lymph nodes. Finally, dendritic cell vaccination combined with Val-boroPro treatment results in complete regression of established tumors. Our findings demonstrate that Val-boroPro has antitumor activity and a novel mechanism of action that involves more robust DC trafficking with earlier priming of T cells. Finally, we show that Val-boroPro has potent adjuvant properties resulting in an effective therapeutic vaccine.

## Introduction

Multiple lines of evidence have conclusively demonstrated that natural immunity can contribute to control of tumor growth [1], [2]. However, once progression occurs, the widely held assumption is that the tumor has evaded the immune response and that the host has become immunologically tolerant [3], [4], [5], [6], [7]. This is particularly important in the context of clinically relevant immune-based therapy where most patients present with established tumor. Multiple mechanisms can contribute to tumor escape, ultimately representing the complex interplay between the tumor and the host immune system [1]. We, and others, have shown that tumors effectively prime T cells toward tumor antigens despite progressive tumor growth, and these T cells are functional when removed from the tumor-bearing host [8], [9]. Thus, the ability to recover productive immune responses once a tumor has become established will be critical for the successful implementation of strategies to augment existing antitumor immunity therapeutically. Priming T cell responses by tumors is dependent on presentation of tumor antigens by bone marrow-derived cells [10] with the relevant subset being a dendritic cell (DC) [11], [12], [13]. Furthermore, this process requires the successful migration of DCs to the secondary lymphoid structures (likely the tumor draining lymph node) where antigen presentation and priming of T cells occurs [9], [14]. Although DCs can infiltrate tumors, the functions of DCs appear to be impaired in tumor-bearing mice and humans [15], [16], [17], [18]. Thus, modulation of antigen presenting cells such as DCs represents a promising approach to reverse suppressive mechanisms and enhance adaptive immune responses toward cancer [19].

Post-proline cleaving enzymes (PPCEs) are a ubiquitous family of serine proteases that selectively cleave after proline or alanine two amino acid residues from the N-terminus of peptides, and hydrolyze a number of known substrates, including multiple chemokines [20]. Inhibitors of one particular PPCE, dipeptidyl peptidase (DPP)IV, have been FDA-approved due to their ability to prevent hydrolysis of the glucagon-like peptide 1 incretin and thereby improve glycemic control [21]. In addition to DPPIV, which is the prototypical extracellular member of the PPCE family associated with CD26 on the cell surface, the family also includes the intracellular proteases, DPP2, DPP8, and DPP9 [22]. Fibroblast activation protein (FAP or seprase) is another PPCE that, similarly to DPPIV is expressed as a cell-surface protein with extracellular proteolytic activity. Unlike DPPIV, however, the expression of FAP appears to be selectively upregulated in non-transformed stromal cells of the tumor microenvironment [23]. Depletion of FAP-expressing stromal cells impairs growth of an immunogenic tumor via a mechanism dependent on lymphocytes and generates successful therapeutic vaccination in established tumors [24]. Inhibition of FAP using extracellular competitive inhibitors of DPPs has been shown to contribute to impaired epithelial cancer growth via a mechanism that is dependent on FAP [25]. The role of extracellular proteases in both the progression and inhibition of cancer has been well documented [26]. However, much less is known about the intracellular PPCEs in the context of tumors.

Dipeptide boronic acids (DBAs) are synthetic compounds which potently inhibit PPCEs [27]. *In vitro*, DBAs can increase production of G-CSF, IL-6, and IL-11 by bone marrow stromal cells, thereby stimulating hematopoietic progenitor cell growth [27]. In addition, one particular DBA, L-valinyl-L-boroproline (Val-boroPro), has been shown to produce potent antitumor effects when administered orally in multiple mouse tumor models [20], [28]. Importantly, the antitumor effects appear to require an intact host immune response, because efficacy was severely reduced in lymphocyte-deficient mice. Analysis of lymphoid tissue and tumors revealed increases in mRNA encoding multiple cytokines and chemokines following Val-boroPro treatment; however, the immunologic mechanism of the tumor response and the extent to which it is dependent upon inhibition of PPCEs has continued to remain uncertain. Using an immunologically well-characterized tumor model, we demonstrate here that Val-boroPro mediates complete tumor regression via a novel mechanism that requires more rapid DC trafficking and subsequent acceleration of T cell priming. Importantly, although FAP has been implicated in regression of epithelial tumors mediated by Val-boroPro and other boronated dipeptides targeting extracellular enzymes, our data suggest that inhibition of intracellular DPPs may also contribute to tumor regression. Finally, we show that when Val-boroPro administration is combined with a DC vaccine, regression of large, established tumors is achieved.

## Materials and Methods

### Mice

C57BL/6 mice and B6CD45.1 congenic mice were purchased from the Animal Production Facility at NCI-Frederick and housed until 6 weeks of age. Rag1^−/−^, C57BL/6-*Il5^tm1Kopf/^*J (IL-5^−/−^), OT-1 TCR transgenic mice, CD11c^+^-diphtheria toxin receptor transgenic mice, CCR7−/− and GFP transgenic mice were purchased from Jackson Laboratories (Bar Harbor, ME). EPO DTP (PHIL) eosinophil-deficient mice were kindly provided by Dr. Jamie Lee [29]. IL-17^−/−^ and IL-23p19^−/−^ mice were kindly provided by Dr. Giorgio Trinchieri (NCI). plt/plt mice with a genomic deletion in Scya19 and Scya21a encoding CCL19 and lymphoid organ-derived CCL21 respectively [30] were kindly provided by Dr. Ester Roffe (NIAID). Animals were kept in a specific pathogen-free facility at the NIH (Bethesda, MD) and handled according to protocols approved by the NCI Animal Care and Use Committee (Assurance Number A4149-01). To minimize suffering, mice were euthanized at predetermined endpoints.

### Tumor Cell Lines

MB49 [8], 76-9 [31], and M3-9-M [32], [33] were cultured in 10% complete mouse media (RPMI-1640 with 10% heat inactivated FCS, 1% HEPES buffer, 1% penicillin/streptomycin/L-glutamine, 1% Non-essential amino acids and 2-mercaptoethanol 50 µM/L) at 37°C in 5% CO_2_ (Invitrogen, Carlsbad CA). Unless otherwise noted, single cell suspensions of exponentially growing MB49 cells (1×10^6^cells/injection) were injected subcutaneously over the flanks of mice on day 0. M3-9-M cells (1×10^6^) or 76-9 cells (5×10^5^) were injected intramuscularly into the gastrocnemius muscle. Secondary MB49 (1×10^6^ cells/injection) was injected subcutaneously on the opposite flank for tumor rechallenge experiments. Tumors were measured in two dimensions (LxW) two to three times a week by digital caliper, and approximate spherical volumes were calculated ((L/2)×(W/2)×[(L+W/2)/2]×4/3π). Mice were euthanized when tumor diameters reached 2 cm, in accordance with animal protocols.

### In vivo Post-proline Cleaving Enzyme Inhibition

Val-boroPro and PT-630 were provided by Point Therapeutics (Boston, MA) and were stored at −80°C. Concentrated aliquots (100 mM) dissolved in 0.1 N HCl were diluted with 1 mL normal saline immediately before use and administered daily by gavage (0.15 mL, 20 µg/dose). Normal saline was administered (0.15 mL/dose) to control animals. Treatment followed a weekly schedule of five days of treatment followed by two days without treatment.

### Immune Assays

ELISPOT assays were performed using IFN-gamma mouse ELISPOT kits according to manufacturer’s instructions (R&D Systems, Minneapolis MN) as previously described [34].

For analysis by fluorescence activated cell sorting (FACS), tissues were made into individual single cell suspensions and red blood cell depleted. FcγIII/II receptors were blocked (2.4G2) and cells were labeled with one of the following antibody panels: (1) CD44-FITC, CD69-PE, CD4-PerCP-Cy5.5, CD8-APC; (2) B220-FITC, NK1.1-PE, CD11b-PerCP, CD11c-APC; (3) CD11b-FITC, CD11c-PE, CD45.2-PerCP, CD45.1-APC; (3) CD62L-FITC, CD11c-PE, CD11b-PerCP, CCR7-APC (BD, Franklin Lakes NJ). Cells were incubated with antibodies at 4°C for 20 minutes, washed, and analyzed in FACS buffer (0.1% sodium azide, 0.5% fetal bovine serum, 2mM EDTA). Flow cytometry was performed using a four channel FACSCalibur® and CellQuest software (BD, Franklin Lakes NJ), and samples were further analyzed using FlowJo software version 8.2 for Macintosh (Tree Star Inc, Ashland OR).

Serum cytokine analysis was performed using a multiplex murine Th1/Th2 kit (Mesoscale) according to manufacturers instructions. IL-17 ELISA (R&D systems, Minneapolis, MN) was performed based on manufacturer’s instructions.

### T Cell Depletion

Monoclonal antibodies to CD4 (GK1.5) and CD8 (2.43) were used to deplete T cells (GTC Washington Laboratories, Gaithersburg MD). Antibodies were administered intraperitoneally (0.5 mg/dose in PBS) three times a week for four weeks, with two doses given prior to tumor inoculation (first dose: 1 mg/injection). Depletion of CD4 or CD8 T cell populations following a 0.5 mg dose of GK1.5 or 2.43 respectively was confirmed by flow cytometry to be >97% at 24 hours.

### Adoptive Transfer of Purified T Cells

Donor C57BL/6 female mice were inoculated with MB49 (1×10^6^ cells) and treated with Val-boroPro or saline for two weeks. On day 17 post tumor injection, spleens and axillary, brachial, and inguinal LNs from Val-boroPro-treated, saline-treated, and naïve C57BL/6 female mice were harvested, pooled based on treatment, and made into single cell suspensions. T cells were purified using Pan T cell isolation kits (Miltenyi Biotech, Auburn CA) and adoptively transferred into Rag1^−/−^ recipients (1×10^7^ T cells/injection). Three days later, mice were challenged with high dose MB49 (3×10^6^ cells/injection) and tumor growth was monitored.

### Macrophage Depletion

To deplete macrophages *in vivo*, clodronate was administered intraperitoneally at a dose of 0.1 mL/10 g on day −1 and every other day from day 2 to day 8 during Val-boroPro treatment (days 3–7). Twenty-four hours after clodronate administration, macrophages were >95% depleted at 16 hours, as confirmed by flow cytometry.

### CD11c^+^ Cell (Dendritic Cell) Depletion

CD11c-diphtheria toxin (DT) bone marrow chimeras were generated by transplanting 5×10^6^ bone marrow cells from CD11c-DT receptor transgenic mice into lethally irradiated (1200 cGy) C57BL/6 female recipients. Following full engraftment at 28 days CD11c^+^ cells were depleted with DT (8 ng/g in phosphate-buffered saline), administered intraperitoneally every other day from day −1 of tumor injections to day 9. Val-boroPro was simultaneously administered during week one of tumor growth (days 3–7). >98% CD11c^+^ cell depletion was confirmed by flow cytometry 24 hours post-injection, with durable depletion observed until three days after the last injection.

### Dendritic Cell Trafficking

Ly5.2 (CD45.1) mice were treated with Val-boroPro or saline for three days. Spleens and axillary, brachial, and inguinal LNs were pooled, harvested, and made into single cells suspensions. CD11c^+^ cells were selected using CD11c^+^ microbead positive selection (Miltenyi Biotech, Auburn CA). 5×10^6^ congenic (CD45.1) CD11c^+^ cells were injected into lateral tarsals of four groups of naïve C57BL/6 (CD45.2) mice. Two groups received cells from Val-boroPro treated mice and two from saline treated animals. One subset from each group received Val-boroPro following DC injections. Fifteen hours post injection, lateral tarsal-draining popliteal LNs were harvested from animals, made into individual single cell suspensions, and analyzed using flow cytometry.

For *in vivo* cell trafficking, tumors were injected with DCs from GFP^+^ mice. Whole tumor-draining lymph nodes were placed onto glass slides and viewed using a Zeiss AxioObserver Z1 microscope (Zeiss Inc., Thornwood NY) using a 4× bright field objective. GFP^+^ cells were detected using a 38HE emission BP 525/50 filter set. Images were captured with a Zeiss AxioCam MRm monochrome digital camera. Several images were required to capture the entire lymph node and the images were then tiled and analyzed using AxioVision 4.6 software.

### Dendritic Cell Vaccines

Dendritic cells were cultured from bone marrow of male or female C57BL/6 mice and maintained for 8 days in 10% complete mouse media supplemented with GM-CSF, IL-4, SCF, Flt-3 and IL-3 (1 µL/mL) (PeproTech, Inc., Rocky Hill NJ) at 37°C and 5% CO_2_ as described previously [35]. Where indicated, DCs were incubated for 24 hours with irradiated whole tumor cells. DCs were activated *in vitro* with anti-CD40 (4 µL/mL media) on day 7, 24 hours before harvesting and were injected intraperitoneally (1×10^5^ cells/injection).

### Statistical Analysis

All statistical analysis was performed using GraphPad Prism software, version 4.0c (GraphPad Software Inc, La Jolla CA). Two-tailed Mann-Whitney tests were used to analyze differences between two treatment groups in ELISPOT assays and FACS analysis. One-way ANOVA (Kruskal-Wallis test and Dunn’s Multiple Comparison test) was used to test significance of tumor volumes between 3 or more groups in tumor growth experiments. For tumor growth analysis with only two groups, two-tailed Mann-Whitney tests were performed on data points from groups on a specific day, as indicated. A value of p<0.05 for all tests was considered significant. Survival curves were analyzed using a Logrank test. Asterisks in figures represent the degree of significance between groups (*p<0.05, **p<0.01, ***p<0.001).

## Results

### Treatment with Val-boroPro as a Single Agent Induces Tumor Regression

**Figure 1:**
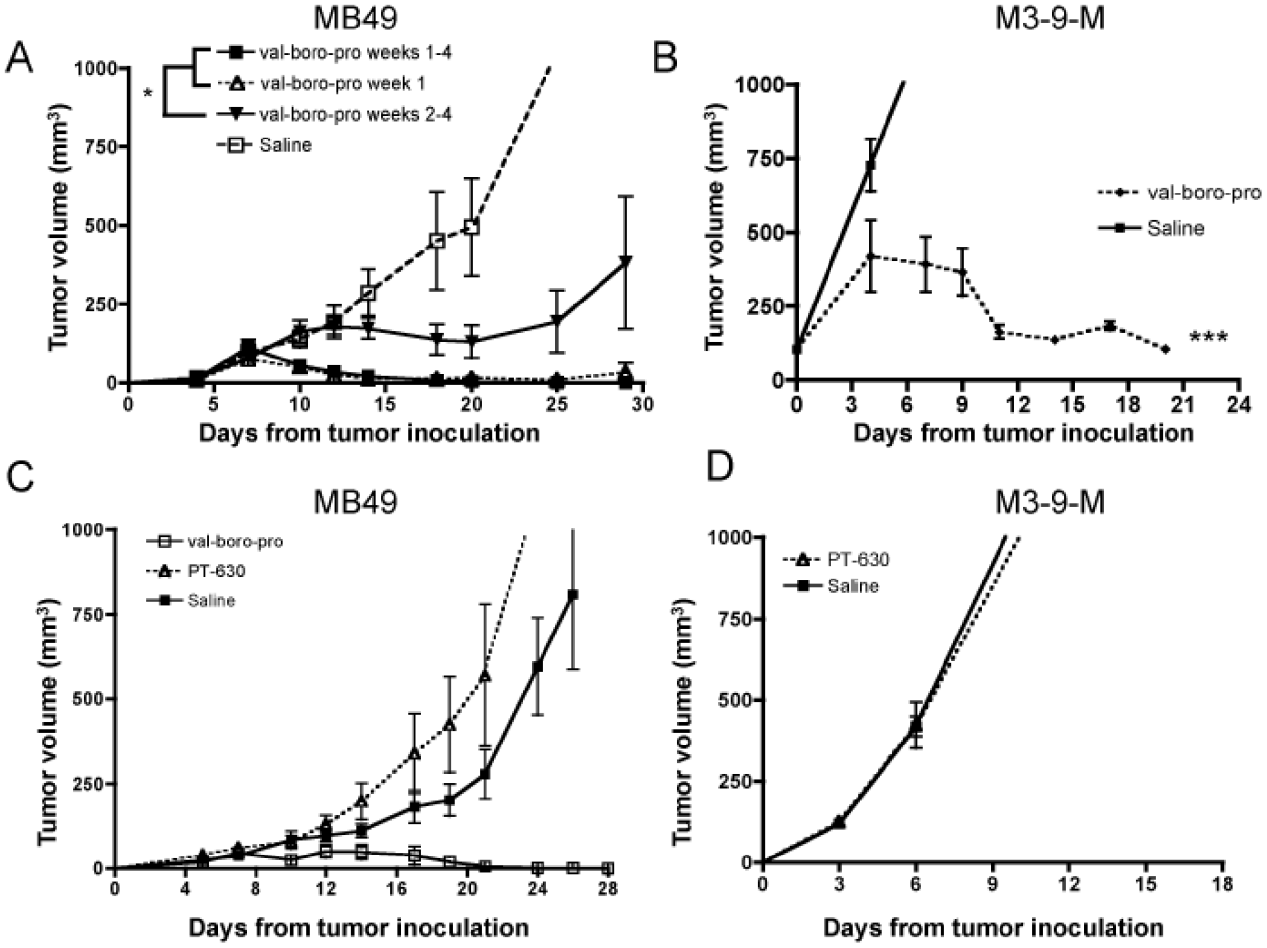
Val-boroPro induces complete regression in multiple tumor models and requires inhibition of intracellular PPCE. (A) C57BL/6 female mice were inoculated with 1×10^6^ MB49 on day 0. Mice were treated with saline (open squares) or 20 µg Val-boroPro 5×/week during weeks 1 through 4 (days 3–28, closed squares), week 1 only (days 3–7, open triangles), or weeks 2 through 4 (days 10–28, inverted solid triangles) (n = 6/group). All treatment groups are statistically different than saline (Anova, p<0.05 for late treament vs saline and p<0.01 for early treatment groups vs saline). The days 10–28 group is also statistically different than both early treatment groups (p<0.05). (B) C57BL/6 mice were inoculated with 1×10^6^ M3-9-M on day 0 and treated with 20 ug Val-boroPro (dashed line) or saline (solid line) for four weeks (n = 5/group). Tumor volumes are statistically different between the Val-boroPro and saline groups at p<0.001 for all times beyond day 10. (C) Mice were inoculated with MB49 as in Figure 1A and treated with 20 ug Val-boroPro (open squares), 20 ug PT-630 (open triangles), or saline (closed squares) (n = 5/group). (D) Mice were inoculated with M3-9-M as in Figure 1B and treated with PT-630 (open triangles) or saline closed squares) (n = 5/group). Mean tumor volumes show that PT-630 treatment was no different from saline. Figures 1A–D are representative of 3 or more experiments.

Val-boroPro administration resulted in complete eradication of the murine bladder carcinoma, MB49 (Figure 1A) following initial progression during the first week of treatment. Despite discontinuation of Val-boroPro at peak tumor size, clearance of tumors still occurred with no recurrence during 50 days of subsequent observation. Antitumor activity was observed when Val-boroPro treatment was delayed until day 10 after tumor injection, but progression and lethality eventually ensued (Val-boroPro weeks 2–4, Figure 1A). Antitumor activity was also observed with 5 days of Val-boroPro treatment following the orthotopic implantation of the murine rhabdomyosarcoma line M-3-9M (Figure 1B). It was previously reported that inhibition of exclusively extracellular proteases with PT-630, a lipophobic amino boronic dipeptide unable to cross the cell membrane, mediates FAP-dependent antitumor activity in a murine colon carcinoma model [25]. However, PT-630 did not demonstrate antitumor activity in either MB49 or M-3-9M (Figure 1C, 1D). Thus, although FAP may be a sufficient target in some murine tumor models, we demonstrate that extracellular enzyme inhibition is insufficient in our models, suggesting intracellular proteases may also be relevant targets for boronated dipeptides with antitumor activity.

### Tumor Eradication Mediated by Val-boroPro is CD4^+^ and CD8^+^ T Cell Dependent

**Figure 2:**
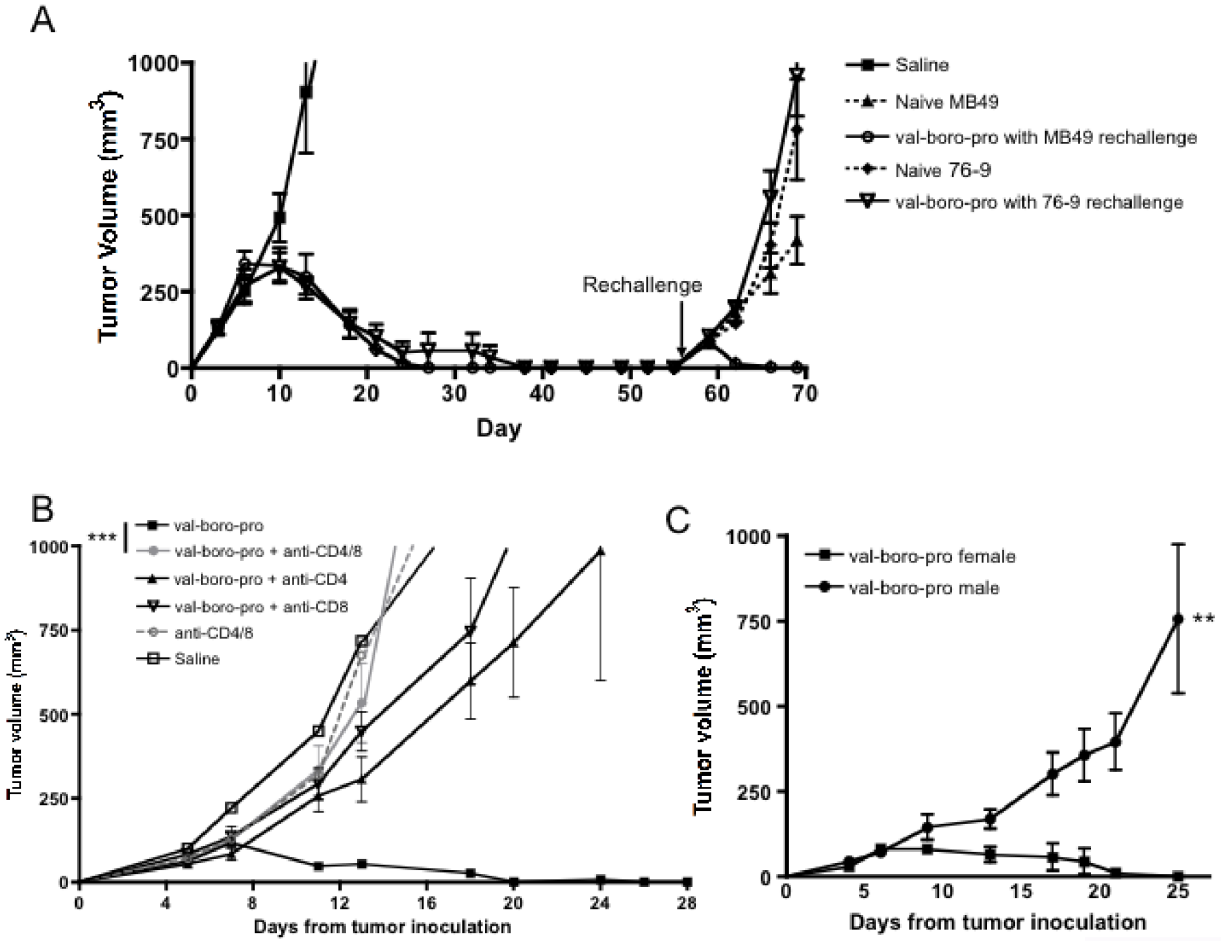
Val-boroPro mediated tumor regression is T cell dependent. (A) Female C57BL/6 mice were challenged on day 0 with 1×10^6^ MB49 and treated with 20 µg Val-boroPro during weeks 1 through 4 (5× per week). Mice that were treated with 20 µg Val-boroPro and subsequently rejected MB49 were then rechallenged with MB49 (1×10^6^) or 76-9 rhabdomyosarcoma (5×10^5^) on day 56 post-primary challenge (n = 5/group). Saline control, initial treatment (closed squares), naïve mice (that did not previously reject tumors) injected with MB49 (closed triangles) or 76-9 (closed diamonds) at time of rechallenge, mice that previously rejected MB49 during Val-boroPro and rechallenged with MB49 (open circles) or 76-9 (inverted open triangles). (B) Mice were inoculated with MB49 as in Figure 1A and treated with Val-boroPro or saline (closed squares) (n = 5/group). CD4^+^ and/or CD8^+^ T cells were depleted with anti-CD4 and/or anti-CD8 monoclonal antibodies as outlined in methods. Tumor volumes in Val-boroPro treated mice without T cell depletion (closed squares) were significantly smaller than either CD4 (closed triangles) or CD8 (inverted open triangles) depleted groups (p<0.05) and CD4/CD8 depleted group (closed circles) (p<0.001) Open circles represent anti-CD4/anti-CD8 without Val-boroPro treatment. (C) Male (closed circles) and female (closed squares) mice were challenged subcutaneously with 10^6^ HY-expressing tumor MB49 on day 0 and were treated with 20 µg Val-boroPro 5×/week for two weeks (n = 5/group). P<0.01 for all timepoints beyond day 15. Figures 2A–C are representative of 3 experiments.

Tumor eradication induced when Val-boroPro was discontinued at maximum tumor size as well as previous data demonstrating lack of efficacy in lymphocyte-deficient mice [20] suggests an immune-mediated mechanism. To confirm that immunologic memory was induced, we rechallenged mice with MB49 on the opposite flank three weeks after eradication of primary tumors by Val-boroPro. All mice rejected the rechallenged tumors after an initial period of tumor growth without any further treatment with Val-boroPro (Figure 2A). Val-boroPro-mediated clearance of MB49 did not protect against secondary rechallenge with the rhabdomyosarcoma line 76-9. These results indicate the priming of adaptive immunity and establishment of tumor-specific immunologic memory in the Val-boroPro-treated animals.

To establish which types of lymphocyte are required for the antitumor response mediated by Val-boroPro, CD4^+^ and/or CD8^+^ T cells were depleted during and following treatment. Complete loss of the antitumor activity of Val-boroPro resulted from depletion of both CD4^+^ and CD8^+^ subsets and nearly complete loss from depletion of either subset alone (Figure 2B). Thus, the eradication of tumors by Val-boroPro is T-cell dependent. The lack of effect in the absence of T cells also confirms that direct effects of Val-boroPro on tumor cell viability are unlikely to contribute to the antitumor activity *in vivo*.

### Val-boroPro Accelerates Tumor-induced CD4^+^ and CD8^+^ T Cell Priming

MB49 expresses well-characterized H2D^b^-restricted CD4^+^ and CD8^+^ epitopes derived from the HY male antigenic complex, and tumor-induced expansion of HY-specific T cells can be measured during tumor progression [8]. There was a transient and modest decrease in the rate of tumor growth in Val-boroPro treated male mice, which are immunologically tolerant to HY, suggesting the possibility of immune response to non-HY tumor antigens; but Val-boroPro did not mediate complete tumor eradication in the male mice (Figure 2C). The result confirms the importance of HY in stimulating tumor immunity in this model and is also consistent with the T cell requirement for Val-boroPro-mediated tumor regression.

**Figure 3:**
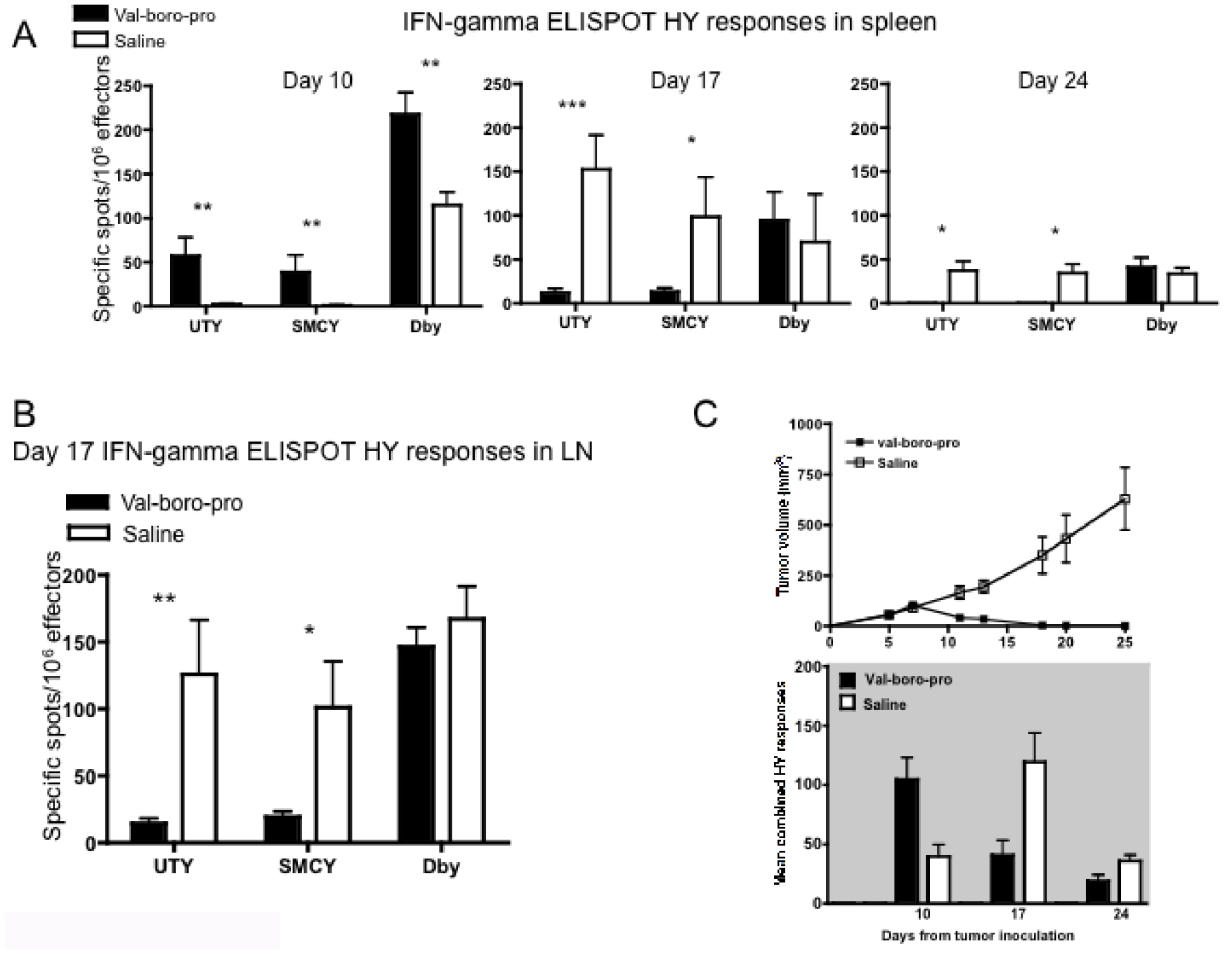
Val-boroPro treatment is associated with an acceleration of tumor-induced priming. (A) Female mice were inoculated with MB49 and treated with Val-boroPro (closed bars) or saline (open bars) (n = 12/group). Spleens and lymph nodes were harvested from mice on days 10, 17, and 24 for IFN-gamma ELISPOT analysis (*p<0.05, **p<0.01, ***p<0.001, Mann-Whitney). (B) Mice were treated as in Figure 3A. On day 17, greater numbers of HY-reactive CD8^+^ T cells were observed in the lymph nodes of saline-treated (open bars) mice compared to Val-boroPro treated (closed bars) mice (*p<0.05, **p<0.01, Mann-Whitney, n = 5/group). (C) Composite data showing combined (UTY+SMCY+DBY) relative to tumor volume from mice treated in figure 1A with saline (open squares top, open bars bottom) or Val-boroPro (closed squares top, closed bars bottom) (n = 12/group). Figures 3A–C are representative of 3 experiments.

We next investigated whether Val-boroPro increases the frequency of tumor-specific T cells. Following MB49 inoculation of female mice, significant increases in HY-specific CD4^+^ and CD8^+^ T cells were observed in Val-boroPro treated animals at day 10 compared to controls (Figure 3A). By days 17 and 24, however, HY-specific cells were also expanded substantially in the spleens and LNs of saline-treated animals with progressive tumors (Figure 3A and 3B). When comparing tumor curves with the composite responses to all HY epitopes (UTY+SMCY+DBY), it is apparent that the peak of the HY-specific T cell response occurs at the onset of tumor regression in Val-boroPro-treated mice and occurs earlier than the peak response in control mice (Figure 3C). Interestingly, the magnitude of the HY-specific T cell response in Val-boroPro treated mice is no higher than that eventually observed at a later stage of tumor growth in the absence of Val-boroPro. We conclude that complete tumor regression mediated by Val-boroPro results from a more rapid expansion of tumor-specific T cells rather than from an increase in the magnitude of the immune response.

### T Cells Primed by Tumors in the Presence of Val-boroPro Demonstrate Increased Potency Following Adoptive Transfer

**Figure 4:**
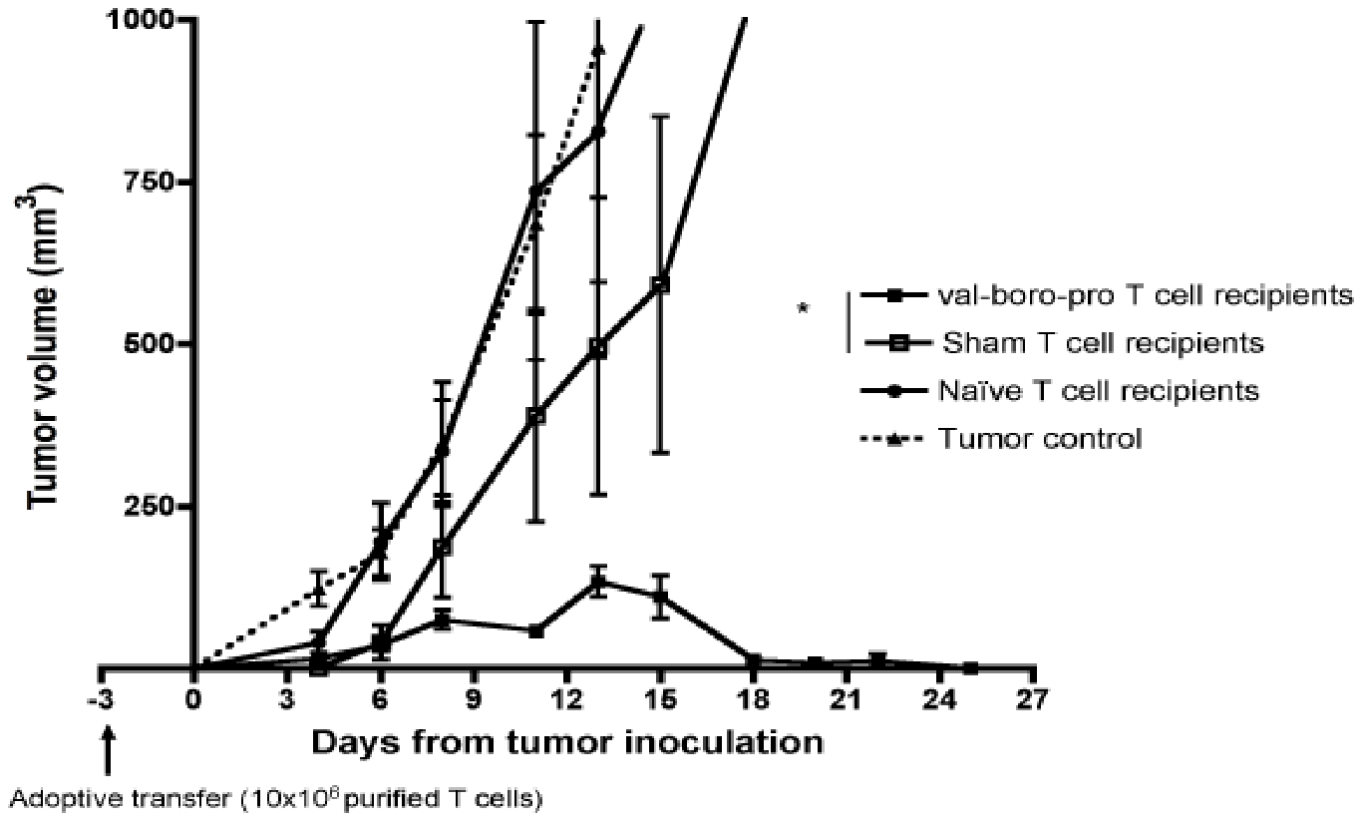
Val-boroPro treatment improves functionality of adoptively transferred T cells. Donor C57BL/6 female mice were inoculated with 1×10^6^ MB49 on day −20 and treated with 20 µg Val-boroPro or saline 5×/week for two weeks. On day −3, purified donor T cells from pooled donor lymph nodes were transferred into Rag1^−/−^ recipients intravenously (1×10^7^/mouse). On day 0, Rag1^−/−^ mice were challenged with high-dose MB49 (3×10^6^). Recipients of T cells from tumor-bearing Val-boroPro treated donors (closed squares) had significantly smaller tumors than in recipients of T cells from tumor-bearing saline treated donors (open squares) (p<0.05 for all timpoints after day 12). Open squares, recipients of T cells from non tumor-bearing donors; closed triangles, Rag −/− mice injected with MB49 but not receiving T cells. Figure 4 is representative of 4 experiments.

We next employed an adoptive transfer model to assess the activity of tumor-primed T cells from Val-boroPro treated mice. Purified T cells from LNs of either naïve mice or tumor-bearing mice treated with Val-boroPro or saline were adoptively transferred into Rag1^−/−^ recipients. Five days later, recipients were challenged with high-dose MB49. Despite reduced frequency of HY specific T cells in Val-boroPro treated tumor-bearing mice at the time of collection (day 17, Figure 3B), T cells from treated donors produced complete tumor eradication and their efficacy was clearly superior to that of T cells from saline-treated donors and (Figure 4). Importantly, the antitumor activity of primed T cells occurred without exposure to Val-boroPro in recipient mice.

### Val-boroPro Increases Serum IL-5 and Induces IL-17 Production by Antigen-primed T Cells but Antitumor Efficacy is not Dependent on IL-5, Eosinophils, IL-17 or IL-23

**Figure 5:**
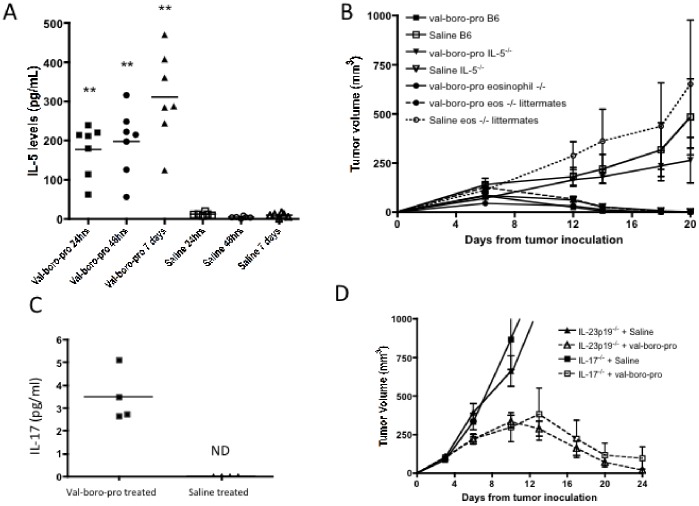
Val-boroPro increase IL-5 and IL-17 production but tumor regression is not dependent on IL-5, IL-17 or IL-23. IL-5 and IL-17 levels increase following Val-boroPro treatment but are not required for antitumor activity. (A) Female C57BL/6 mice were treated with 20 µg Val-boroPro or saline, and serum was collected from mice after 24 hours, 48 hours, and 7 days of treatment (n = 7/group). Serum samples were analyzed by cytokine multiplex assay. At all time points, Val-boroPro treated mice had significantly higher levels of IL-5 (pg/mL) than saline treated controls (p<0.01 at all timepoints, Mann-Whitney). (B) Eosinophil^−/−^ (PHIL), IL-5^−/−^, and eosinophil^+/−^ littermates were challenged with MB49 (10^6^) on day 0 and treated with Val-boroPro or saline for 1 week (days 3–7) (n = 5/group). There was no statistical difference between any of the Val-boroPro treated groups. (C) Tumor-free OT-1 TCR transgenic mice were treated with 20 ug Val-boroPro or saline for 3 days (n = 4/group). One hour following the third dose, spleens were harvested and T cells were cultured and stimulated with 10 µM OT1 peptide. Supernatants were harvested after 24 hours and analyzed for IL-17 secretion by ELISA. IL-17 was not detected (ND) in T cell cultures from saline treated mice. (D) IL-17 or IL-23p19^−/−^ mice were inoculated with MB49 (1×10^6^) and treated with Val-boroPro or saline for 2 weeks (n = 7/group). Experiments displayed in Figures 4A–D were each conducted twice.

It has been previously shown that Val-boroPro alters the protein expression of several cytokines and chemokines [20]. Using a multiplex assay, we observed increased IL-5 as early as 24 hours following Val-boroPro treatment (Figure 5A) with no changes in other cytokines measured (IFNγ, TNFα, IL-1β, IL-2, IL-4, IL-10, or IL-12). However, Val-boroPro antitumor activity was preserved in both IL-5 deficient mice and eosinophil deficient (PHIL) mice (Figure 5B). It has recently been shown that mice with T cells deficient in DPP2 generate predominantly Th17 cells [36]. Additionally, we found that TCR transgenic CD8^+^ T cells from Val-boroPro treated mice produced more IL-17A following peptide stimulation *in vitro* (Figure 5C). However, tumor-bearing mice deficient in IL-17 or the Th17-associated cytokine, IL-23 remained capable of rejecting tumors with Val-boroPro treatment (Figure 5D). Thus, although changes in cytokines were apparent following Val-boroPro-treatment, IL-5, IL-17 and IL-23 are not required for antitumor activity.

### Val-boroPro-mediated Antitumor Activity is Dependent on Dendritic Cells

**Figure 6:**
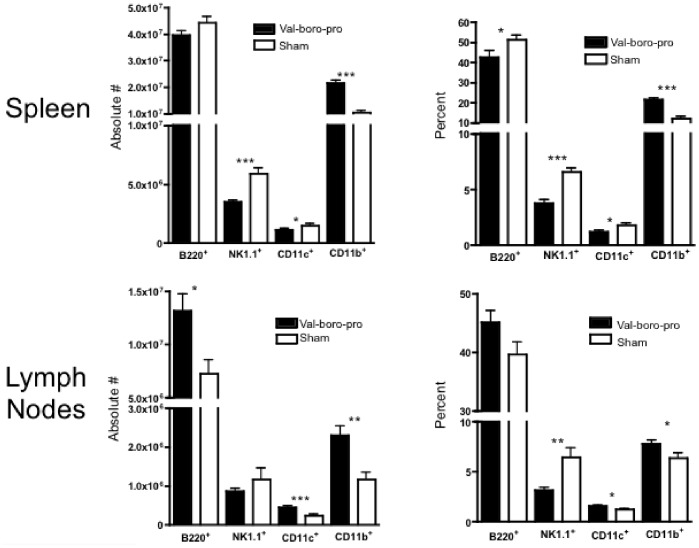
Treatment with Val-boroPro alters lymphocyte and myeloid cell population in secondary lymphoid tissues. C57BL/6 mice were treated with 20 µg Val-boroPro (solid bars) or saline (open bars) 5×/week for four weeks, and spleens (n = 15/group) and lymph nodes (n = 8/group) were harvested and analyzed by flow cytometry. (*p<0.05, **p<0.01, ***p<0.001 and reflect comparisons between Val-boroPro treated and Saline treated groups by Mann-Whitney). Figure 6 was conducted twice.

**Figure 7:**
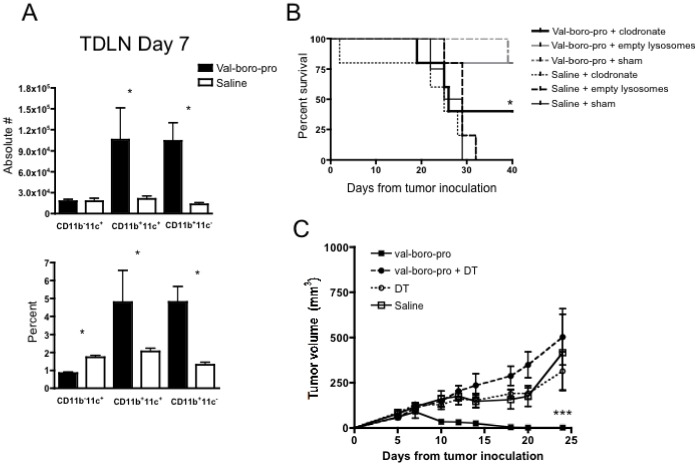
Myeloid dendritic cells (CD11b^+^CD11c^+^) are increased in TDLN and are required for tumor regression. (A) C57BL/6 mice were challenged with 1×10^6^ MB49 on day 0 and treated with Val-boroPro (closed bars) or saline (open bars) during week one (n = 4/group). Tumor-draining lymph nodes were harvested and analyzed by flow cytometry on day 7 (p<0.05, Mann-Whitney) (B) C57BL/6 mice were inoculated with 10^6^ MB49 and injected with clodronate IP (0.1 mL/10 g body weight) every other day from day –1 to 9 following tumor inoculation. Control groups were injected with empty lysosomes or saline (sham). Val-boroPro was administered orally for 1 week (days 3–7). Survival was significantly different in Val-boroPro treated mice given clodronate (thick solid line) compared to those treated with Val-boro-Pro and given empty lysosomes (gray solid line) (*p<0.05, Logrank test, n = 5/group). (C) CD11c-diphtheria toxin (DT) chimeric mice were generated by transplanting bone marrow from CD11c-DT transgenic mice into lethally irradiated C57BL/6 recipients (n = 5/group). Female chimeras were inoculated with 10^6^ MB49 on day 0 and treated with Val-boroPro or saline during week one (days 3–7) with or without IP injections of DT (8 ng/1 g body weight) every other day from day –1 to 9. Tumor volumes were significantly larger in Val-boroPro treated chimeric mice receiving DT (closed circles) compared to Val-boro-Pro treated chimeric mice receiving saline (closed squares) (p<0.001 for all timepoints beyond day 10). Tumor volumes were not statistically different between saline treated (open squares) and DT treated (open circles) chimerics not treated with Val-boroPro. Experiments displayed in Figures 7A–C were each conducted twice.

To further examine the mechanism involved in tumor rejection by Val-boroPro we next evaluated changes in immune cell subsets in secondary lymphoid tissues. While B220^+^ cells, CD11c^+^ cells and NK 1.1^+^ cells decreased in both frequency and absolute number in spleens of mice treated with Val-boroPro, there was a marked increase in CD11b^+^ cells (Figure 6). In tumor draining lymph nodes (TDLN) there was a significant increase in CD11b^+^ cells represented by an increase in both CD11c^+^ (myeloid DCs) and CD11c^-^ subsets (Figure 7A). Based on these findings, we investigated whether phagocytic cells were required for antitumor activity. Indeed, as shown in Figure 7B, clodronate treatment, which depletes cells with phagocytic capacity, partially prevented Val-boroPro-mediated tumor regression. Furthermore, DCs appeared to be essential for tumor eradication because selective depletion of CD11c^+^ cells in CD11c-diphtheria toxin receptor bone marrow chimeras completely abrogated the antitumor effects Val-boroPro (Figure 7C).

### Val-boroPro Accelerates Trafficking of DCs to the Tumor-draining Lymph Nodes

**Figure 8:**
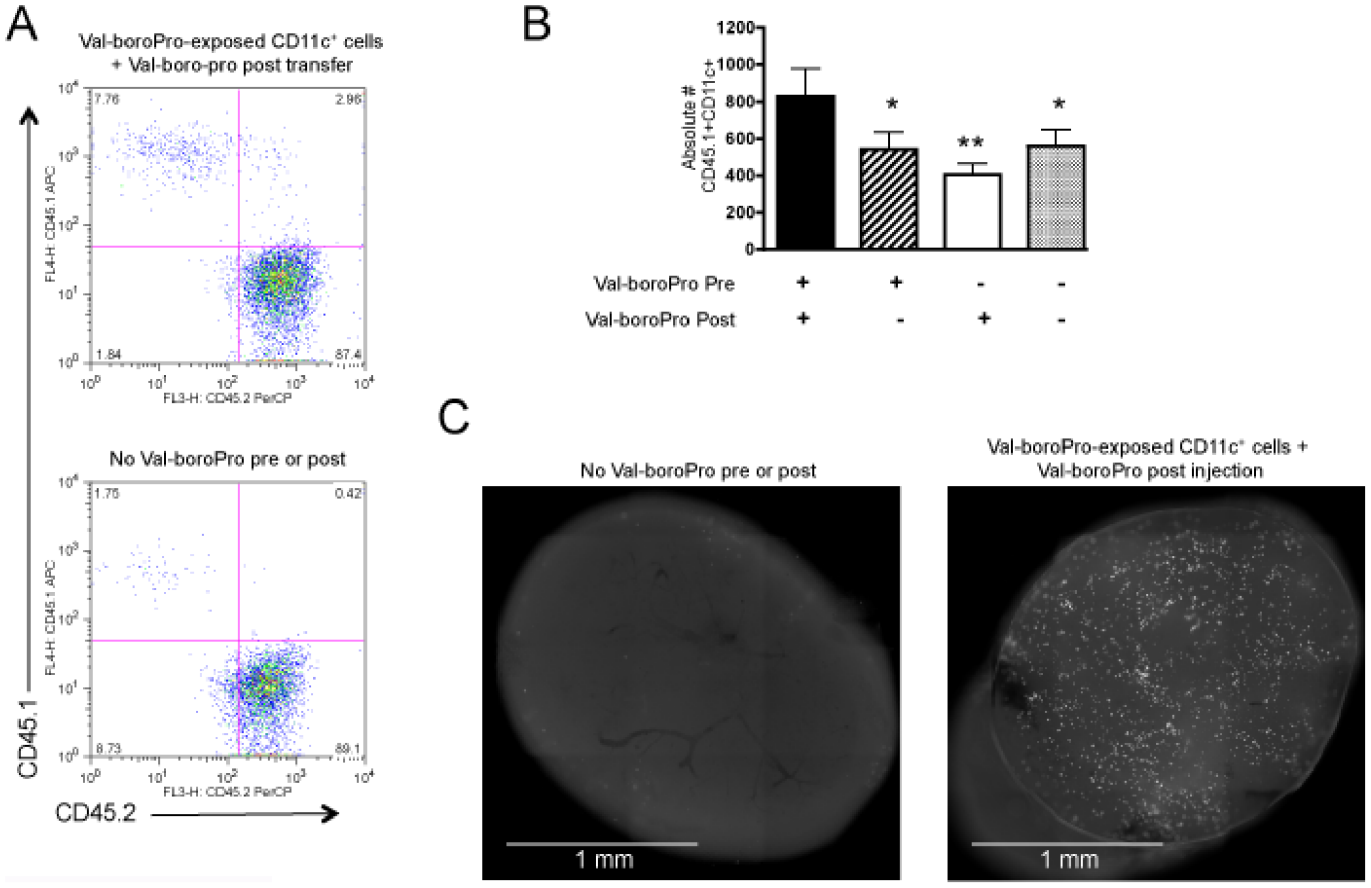
Antitumor activity with Val-boroPro is associated with increased trafficking of DCs. (A–B) B6CD45.1 congenic mice were treated with 20 µg Val-boroPro for three days, and CD11c^+^ cells from spleens and LNs were magnetic bead purified and injected at a dose of 5×10^6^ into lateral tarsals of C57BL/6 (CD45.2^+^) recipients. Immediately following CD11c^+^ cell injection, recipients were treated with Val-boroPro or saline (n = 3/group). Popliteal lymph nodes were harvested 15 hours following Val-boroPro treatment for flow cytometric analysis. (A) Representative dot plots. (B) Bar graph showing statistically more CD45.1^+^ adoptive transferred DCs in the popliteal LN in recipients of DCs from Val-boroPro treated donors also receiving a single injection of Val-boroPro compared to all other group (*p<0.05, **p<0.01, Mann-Whitney). (C) Purified CD11c^+^ cells from Val-boroPro treated GFP^+^ mice were injected intratumorally into established MB49 tumors. Mice were treated immediately thereafter with one dose of Val-boroPro (n = 3/group). Tumor-draining inguinal lymph nodes were harvested 15 hours later and imaged by fluorescent microscopy. Representative lymph nodes from mice receiving CD11c^+^ cells from Val-boroPro treated donors and Val-boroPro post injection. Experiments displayed in Figures 8A–C were conducted 2 times.

We next evaluated whether DC trafficking contributed to the antitumor activity of Val-boroPro. Fifteen hours following lateral tarsal injection of purified CD11c^+^ cells from Val-boroPro-treated donors, enhanced cell trafficking to the popliteal LN was observed compared to that of CD11c^+^ cells from untreated donors (Figure 8A and 8B). This effect was only observed when DCs were exposed to Val-boroPro before and after cells were injected into recipient mice. Next, we directly measured DC trafficking from tumor to TDLN by injecting green fluorescent protein (GFP) expressing (GFP^+^) CD11c^+^ cells from Val-boroPro treated GFP-transgenic donors intratumorally and imaging whole TDLN 15 hours later. As shown in Figure 8C, there was a marked increase in the number of GFP^+^ DCs in the TDLN of Val-boroPro treated recipients injected with DCs from Val-boroPro treated donors.

**Figure 9:**
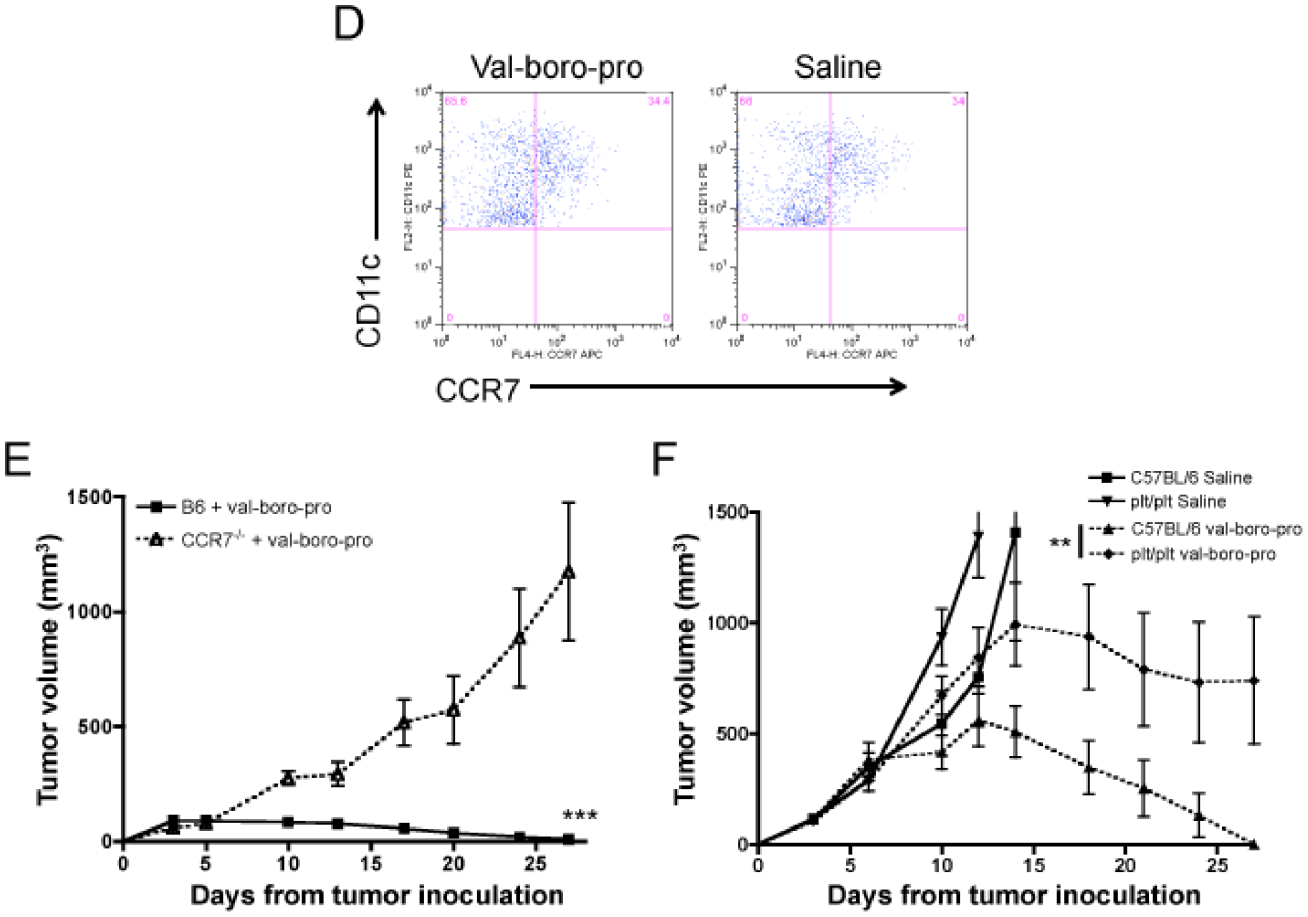
Val-boroPro-mediated acceleration of DC trafficking depends, in part, on the CCR7-CCL19/21 chemokine axis. (A) No change in CCR7 expression on DCs from Val-bor-Pro treated mice. Representative dot plots of gated CD11c^+^CD11b^+^ cells from MB49-bearing mice that were treated with Val-boroPro or saline during week one (days 3–7) and the first day of week two. Mice were sacrificed on day 10 and TDLN were harvested and stained for flow cytometry. (B) CCR7^−/−^ chimeric mice were generated by transplanting T cell depleted bone marrow (5×10^6^) from CCR7^−/−^ mice into lethally irradiated C57BL/6 recipients on day −30. C57BL/6 T cells (CCR7^+^, 1×10^7^) were administered to both groups on day −16, and mice were challenged with 10^6^ MB49 on day 0. CCR7^−/−^ chimeras (open triangles) and C57BL/6 transplanted controls (closed squares) were treated with 20 µg Val-boroPro 5×/week for two weeks (n = 5/group). Tumor volumes were significantly larger in CCR7−/− chimerics receiving Val-boroPro compared to treated recipients of C57BL/6 wildtype bone marrow. p<0.001 at all timepoints beyond day 10. (C) Female C57BL/6 mice and plt/plt mice were inoculated with MB49 (10^6^) on day 0 and treated with Val-boroPro or saline 5×/week for 2 weeks (n = 5/group). Val-boroPro-treated plt/plt mice (closed diamonds) had significantly larger tumors than treated C57BL/6 wildtype mice. P<0.01 at all timepoints beyond day 15. Figures 6A–C are each representative of 3 or more experiments. Experiments in Figures A–C were conducted 2 times.

Trafficking of DCs to the TDLN is primarily mediated by the chemokines CCL19 and CCL21, which are ligands for CCR7 [37]. CCR7 expression was equivalent on DCs from Val-boroPro-treated and saline-treated C57BL/6 mice (Figure 9A) as was the serum level of CCL19 and CCL21. To further explore the contribution of this chemokine axis in tumor eradication mediated by Val-boroPro, we created CCR7^−/−^ bone marrow chimeras reconstituted with CCR7-expressing wildtype T cells to exclude T cell trafficking effects. Following inoculation of MB49, Val-boroPro effectively induced tumor eradication in recipients of wildtype bone marrow but not in CCR7^−/−^ chimeras (Figure 9B). We then tested Val-boroPro in plt/plt mice, which lack functional CCL19 and CCL21. As shown in Figure 9C, a significant difference in tumor volumes was observed between wildtype and plt/plt mice treated with Val-boroPro, but antitumor activity was not completely abrogated in plt/plt mice. In composite, these findings are consistent with a requirement for DC trafficking in the antitumor activity of Val-boroPro but suggest other chemokines besides CCL19/CCL21 may be involved.

### Generation of a Therapeutic Vaccine in an Established Tumor Model

**Figure 10:**
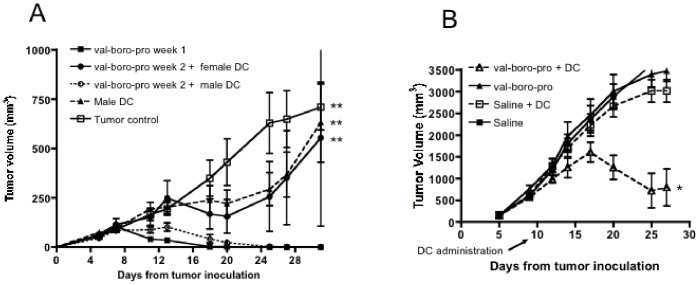
Val-boroPro is an effective adjuvant to tumor primed dendritic cell vaccination. (A) C57BL/6 female mice were challenged with MB49 (1×10^6^) on day 0 and treated with 20 µg Val-boroPro or saline from days 3–7 (closed squares) or 10–14 (n = 5/group). On day 12, late-treatment groups received either a male-derived HY-expressing DC vaccine or a female-derived DC vaccine IP (1×10^5^). Tumors were significantly smaller in mice receiving male DC vaccine plus Val-boroPro during week 2 (open circles) compared to no treatment (open squares), male DC alone (closed triangles) or female DC plus Vale-boroPro during week 2 (closed circles) (p<0.01, Anova with Tukey post-test) (B) C57BL/6 mice were injected intramuscularly with M3-9-M and treated with Val-boroPro starting on day 10. Bone marrow-derived DCs pulsed with irradiated M3-9-M were given IP on day 12 (n = 8/group). Tumors were significantly smaller in mice receiving combined treatment (open triangles) compared to no treatment (closed squares) or either treatment alone (open squares or closed triangles) (p<0.01, Anova). Figures 7A and 7B are both representative of 2 experiments.

Given the impact of Val-boroPro on DC trafficking, we hypothesized that this agent could act as a novel and potent adjuvant to tumor-directed DC vaccination. As shown in Figure 1A, delayed Val-boroPro treatment (Val-boroPro weeks 2–4) of mice with established tumors resulted in transient reduction in tumor growth but was insufficient to induce complete regression. Additionally, despite expression of HY antigens on MB49, vaccination with HY-expressing male DCs alone does not mediate a therapeutic antitumor effect in established tumors (Figure 10A). However, mice given HY vaccine in combination with Val-boroPro demonstrated complete tumor regression despite the large tumor size at the initiation of treatment (day 10). Furthermore, combining a DC vaccine loaded with apoptotic tumor cells and Val-boroPro treatment was also effective in the rhabdomyosarcoma model, M3-9-M [33] (Figure 10B). Again, neither therapy was effective alone against well-established tumors. From these findings, we conclude that Val-boroPro can serve as a potent adjuvant to DC vaccination for treatment of established, bulky tumors.

## Discussion

Immunotherapy represents a promising adjunct to standard cytotoxic and surgical treatment of cancer. Multiple immune-based treatment modalities are being tested in clinical trials, including adoptive cell therapies, monoclonal antibodies and cancer vaccines (reviewed in [38]). Although tumor antigen-specific methods such as adoptive cell therapy have been the most successful at eradicating measurable tumor [39], this approach assumes that optimal targets have been identified for a given patient’s tumor. This is particularly limiting in rare malignancies like pediatric tumors where targets are not well characterized. Vaccines have the advantage of amplifying existing antitumor immune responses and have demonstrated success in the setting of minimal tumor burden [40]. However, methods to increase potency are clearly needed [41]. Using a well-characterized tumor model with known tumor antigens, we confirm a prior report that lymphocyte-dependent tumor eradication can be mediated by Val-boroPro [20] and demonstrate for the first time that this results from a novel mechanism involving acceleration of the process by which a tumor naturally primes T cells. We further establish that this effect is dependent on DCs and is associated with changes in DC trafficking. Finally, we demonstrate complete regression of large, established tumors when Val-boroPro is combined with tumor-specific DC vaccines in two models.

Based on efficacy in animal models, Val-boroPro entered phase I clinical trials in humans [42], [43] in which the compound appeared to be well tolerated and some activity was seen. In subsequent phase II trials in combination with standard cytotoxic chemotherapy, however, Val-boroPro did not meet the endpoints for efficacy [44], [45], . Based on the data presented here, we propose that Val-boroPro and other similar compounds with anti-tumor activity would be far more likely to produce clinical tumor responses when used as immunotherapeutic adjuvants rather than in combination with cytotoxic agents. Indeed, in addition to this report demonstrating for the first time that Val-boroPro can produce regression of large tumors when given with DC vaccines, the compound has also shown an adjuvant effect in a viral vaccine model [47]. Furthermore, the inflammatory profile induced by Val-boroPro [20] and the need for an intact host immune system for full antitumor activity suggests that cytotoxic agents might actually impair clinical activity.

A previous study demonstrated that FAP-expressing tumor-associated stromal fibroblasts contribute to suppression of immune responses in the tumor microenvironment and that ablation of this stromal population can elicit tumor killing by tumor antigen-specific T cells [24]. In a separate study, pharmacological inhibition of the extracellular PPCEs, DPPIV and FAP, appeared to slow tumor growth in lung and colon cancer models [25]. Consequently, FAP and DPPIV are implicated in promoting tumor growth and suppression of tumor immunity might be involved in the mechanism of action of FAP. However, by using PT-630, which is lipophobic and preferentially inhibits extracellular PPCEs, we provide evidence that FAP and DPPIV inhibition might not be sufficient to produce regression in all tumors. It is possible that the contribution of FAP to immune suppression mighty vary depending on the tumor type. Most importantly, our data suggest that other targets besides DPPIV and FAP are involved in the mechanism of tumor regression mediated by boronic dipeptides with antitumor activity such as Val-boroPro. Possible candidates include intracellular peptidases such as DPP2, DDP8, and DPP9. Further studies are needed to fully establish the enzymes targeted by these agents in cancer immunotherapy. However, we conclusively show that the antitumor activity is immune mediated, T cell dependent, requires DCs and is likely to involve accelerated DC trafficking and T cell priming.

There are a number of possible mechanisms by which Val-boroPro treatment could impact DC trafficking, a process that is critically dependent on chemokines. In order for activated DCs to enter the lymph node, they upregulate chemokine receptors and subsequently home to the lymphatic vessels and lymph nodes in response to a chemotactic gradient. Previous work has shown that the CCR7-CCL19/CCL21 axis is a major contributor to this process [48], [49]. Cytokine substrates of PPCEs inhibited by Val-boroPro can be predicted by the presence of either proline or alanine in the position penultimate to the amino-terminal amino acid in the biologically active form of the peptide. There are multiple chemokines with PPCE cleavage sequences in the active forms of chemokines, some of which have already been shown to be truncated by DPPIV with resultant modulation of function [50], including reduced chemoattraction of phagocytes [51]. Thus, it is quite plausible that the enhanced DC trafficking observed in Val-boroPro-treated mice involves potentiation of the chemotactic activity of chemokines *in vivo*. Additional studies are ongoing to define the specific chemokines involved in the DC-trafficking effect. DPPIV is located on the cell surface but tumor regression did not occur in our models with an inhibitor selective for extracellular PPCEs, suggesting that additional changes, perhaps intrinsic to dendritic cells and mediated by intracellular PPCEs such as DPP8 or DDP9, may contribute to the enhanced DC trafficking. This is further supported by the fact that enhanced trafficking was only observed when DCs were exposed to Val-boroPro prior to transfer. It is also possible that other targets besides DPP enzymes are involved in enhanced DC trafficking seen with PPCE inhibition. Regardless of the mechanism by which DC trafficking is impacted by Val-boroPro, the synergistic success of combining Val-boroPro with tumor-specific vaccination against a more established tumor support the use of this agent or similar agents as novel and rationale adjuvants to cancer vaccines.

There are numerous strategies by which tumors evade immune recognition (reviewed in [6], [52]). In the MB49 model, substantial numbers of tumor antigen-specific T cells are expanded in tumor-bearing animals, but this expansion is either insufficient in quantity or pace to reject tumors. The data presented here demonstrate that endogenous tumor priming occurring in the presence of Val-boroPro treatment results in tumor eradication and that T cells from these mice retain potency upon adoptive transfer. Importantly, this does not require an increase in the magnitude of the T cell response but rather an acceleration in the development of the peak immune response. The results suggest that T cell responses to tumor antigens may not always be inherently suppressed, and that it is the pace of T cell priming and thus the magnitude of the T cell response relative to the size of the tumor that is important in determining whether the tumor-specific T cells are effective in killing the tumor.

In summary, our results demonstrate that the boronic dipeptide Val-boroPro induces T cell-mediated tumor rejection via a novel mechanism that involves accelerated T cell priming and enhanced DC trafficking. The end result is that, in the presence of Val-boroPro, tumor-induced T cell tolerance that is often observed with tumor progression is overcome or prevented, resulting in the preservation of T cell function, which can be demonstrated upon adoptive transfer of the T cells. Combining Val-boroPro with a DC vaccine can generate a successful therapy capable of mediating regression of established tumors. Thus, Val-boroPro represents a novel class of agent for boosting endogenous antitumor immune responses. The agent appears to be particularly well suited to clinical application in the setting of immunotherapy with tumor vaccines.
